# Can Psychophysics Be Fun? Exploring the Feasibility of a Gamified Contrast Sensitivity Function Measure in Amblyopic Children Aged 4–9 Years

**DOI:** 10.3389/fmed.2020.00469

**Published:** 2020-08-26

**Authors:** Doaa Elfadaly, Sahar Torky Abdelrazik, Peter B. M. Thomas, Tessa M. Dekker, Annegret Dahlmann-Noor, Pete R. Jones

**Affiliations:** ^1^Moorfields Eye Hospital NHS Foundation Trust, London, United Kingdom; ^2^Department of Ophthalmology, Faculty of Medicine, Minia University, Minia, Egypt; ^3^NIHR Biomedical Research Centre for Ophthalmology, Moorfields Eye Hospital NHS Foundation Trust and UCL Institute of Ophthalmology, London, United Kingdom; ^4^Child Vision Laboratory, Institute of Ophthalmology, University College London (UCL), London, United Kingdom; ^5^Division of Optometry and Visual Sciences, City, University of London, London, United Kingdom

**Keywords:** amblyopia, psychophysics, children, contrast sensitivity function, QUEST+, quick CSF, OpenFace, gabor

## Abstract

Routine assessments of the Contrast Sensitivity Function [CSF] could be useful for the diagnosis and monitoring of amblyopia. However, current CSF measures are not clinically practical, as they are too slow, too boring, and too uncomfortable to sustain a young child's interest. Here we assess the feasibility of a more gamified approach to CSF testing, in which a maximum likelihood psychophysical algorithm (QUEST+) is combined with a largely unconstrained user interface (no fixation target, head restraints, or discrete trials). Twenty-five amblyopes (strabismic, anisometropic, or mixed) aged 4.0–9.2 years performed the gamified CSF assessment monocularly (once per eye). The test required the child to “pop” (press) grating stimuli as they “bounced” around a tablet screen. Head tracking via the tablet's front-facing camera was used to adjust for variations in viewing distance *post hoc*. CSFs were fitted for each eye, and Area Under the CSF (AUCSF) computed as a summary measure of sensitivity. The results showed that AUCSF measurements were able to separate moderately and severely amblyopic eyes from fellow eyes (case-control effect), and to distinguish individuals with varying degrees of vision loss (dose effect). Even the youngest children exhibited no difficulties completing the test or comprehending what to do, and most children appeared to find the test genuinely enjoyable. Informal feedback from a focus group of older children was also positive, although potential shortcomings with the present design were identified. This feasibility study indicates that gamified, child-friendly vision assessments have promise as a future means of pediatric clinical assessment. Such measures could be particularly valuable for assessing children outside of conventional eye-care facilities (e.g., home-monitoring, school screening).

## Introduction

Precise measures of spatial vision (acuity, contrast sensitivity) are important for the diagnosis and monitoring of amblyopia. Ideally, the entire spatial contrast sensitivity function (1) [CSF] should be measured, since sensitivity to low spatial frequency information can be affected in amblyopia, independent of acuity (2–4). Unfortunately, there is at present no effective clinical solution for the routine assessment of CSFs in young children.

One key difficulty is that conventional CSF assessments are too slow to be performed routinely in young children. Thus, while letter charts [e.g., Pelli Robson charts (5)] can provide a rapid summary measure of overall contrast sensitivity, to measure contrast detection thresholds precisely, and to do so across multiple, specific spatial frequencies, typically requires a protracted psychophysical procedure composed of several hundreds of trials (10 min) (6).

Recently, the problem of long test durations has been mitigated by the development of more efficient psychophysical algorithms, such as the “quick CSF” (qCSF) (7–12) or QUEST+ (13, 14). These “maximum likelihood” (ML) algorithms evaluate all previous trials, along with all possible outcomes to any subsequent stimulus, in order to determine the most informative stimulus to present next. This makes ML assessments faster than conventional psychophysical procedures [e.g., adaptive staircases (15, 16)], allowing the whole CSF to be measured in around 30–100 trials (3–10 mins) (6, 8, 10). Furthermore, in situations such as home monitoring, where the same individual undergoes repeated testing, data from any previous assessments can be entered as “prior information,” further reducing any subsequent test durations.

However, while ML assessments are faster than conventional psychophysical methods, current implementations remain inappropriate for the routine assessment of young children. They are often uncomfortable due to the use of chin rests and fixation targets. Furthermore, children often perceive the tests as boring: consisting as they do of a protracted sequence of monotonous, independent “trials” — each following an identical and highly regimented format (e.g., “was the target on the left, or the right?”).

Most adults and older children are willing to tolerate a degree of boredom or discomfort. In younger children, however, such “human factors” can lead to a loss of motivation, often resulting in visual function being critically underestimated (17, 18). Furthermore, even with close monitoring and constant encouragement, it is not uncommon for psychophysical procedures to have to be abandoned in young children. And while high attrition rates, aberrant data points, and extensive supervision can sometimes be accommodated in scientific research, none is sustainable clinically.

In principle, CSF assessments could be made more fun and engaging by adopting a “gamified” approach to vision testing. For example, in the present study we invited children to “pop” bubbles (Gabor patches) by pressing them as they “bounced” around a tablet screen: a task that even very young children tended to find intuitive and engaging (and a response mechanism that forms the basis of many commercial, tablet-based games, targeted at young children). Other research groups are also exploring similar “gamification” strategies. For example, Bosten et al. (19) describe a tablet-based test to screen for color vision deficiency (CVD) in preliterate children (2–6 years), in which the child reveals characters by correctly selecting colored targets. While, Hosokawa et al. (20) have developed a battery of game-like tests designed to probe various visual functions in a hospital waiting area (contrast sensitivity, visual fields, crowding, multiple object tracking).

It is important to recognize, however, that gamification carries a potential cost in terms of empirical rigor. Thus, in designing the test the way we did, we knowingly introduced many potential confounds into our CSF measure, including: criterion effects (i.e., the level of confidence observers felt was necessary before responding); viewing strategies (e.g., whether the user actively scanned the scene or fixated passively in one region); perceptual crowding effects between the multiple targets [i.e., targets shown close together can be harder to detect (21)]; response error (e.g., children pressing too slowly, or in the wrong location); and various stimulus artifacts (e.g., due to screen non-uniformities, variations in viewing angle, or smudges on the screen from repeated pressing).

All of these factors are potential sources of measurement error. Whether they actually prevent the collection of useful data is, however, an empirical question. It may be that a less constrained, gamified procedure is simply unable to produce sensible CSF measurements. At the other extreme, it may be that conventional psychophysical assessments are limited almost entirely by the child's interest and concentration, in which case a gamified test may produce *more* accurate and reliable data than a conventional assessment. Finally, the truth may lie somewhere in between these two extremes – with a gamified test providing a tolerable loss of accuracy, that may be offset by more practical benefits (e.g., higher completion rates or ease-of-use).

To begin to explore the feasibility of a gamified CSF assessment we invited 25 young children (4.0–9.2 years) with diagnosed amblyopia to complete a novel assay that we informally dubbed the “pop CSF” (pCSF) test (see *Methods* for test details). We also asked an advisory group of children and young people with lived experience of various eye and vision conditions to give informal feedback on the test, and to consider its potential pros and cons.

The goal, at this preliminary stage, was not to formally validate a new test or medical device, but to investigate the potential merit of a more gamified approach to psychophysics. In particular, we examined whether children actually found such a test fun and engaging (“usability”), and whether it is capable of producing sensible results (“accuracy”). If so, this would provide grounds for formally validating such a test in a larger sample of amblyopic children in future.

Amblyopia constituted a particularly good test case because, while there is no “gold standard” CSF measure, it is non-contentiously the case that the fellow eye should present as more sensitive (higher CSF) than the affected eye (“case-control effect”) and that children with more severe amblyopia should exhibit a greater between-eye difference in their CSF than children with less severe amblyopia (“dose effect”).

## Methods

### Participants

Participants were 25 children aged 4.0–9.2 years (median {iqr} age 5.8 {1.6} years), with an established clinical diagnosis of strabismic (*N* = 7), anisometropic (*N* = 9) or mixed amblyopia (*N* = 9). Participants were classified as severely (> 0.6 logMAR), moderately (0.3 – 0.6 logMAR), or mildly (0.2 – 0.3 logMAR) amblyopic, based on best-corrected logMAR acuity in their most affected eye. Acuity was assessed by crowded Kay optotypes (<5.0 years) or Thompson crowded letter chart (≥ 5.0 years).

Participants were required to have an interocular difference in best-corrected visual acuity (BCVA) of 0.2 logMAR or greater (22), and to be receiving or starting treatment (either patching or atropine). Exclusion criteria were: (1) other ocular abnormalities; (2) neurological abnormalities including cerebral visual impairment; (3) developmental disorders; or (4) deprivation amblyopia.

Children were recruited from children's clinics (orthoptic and consultant-led clinics at Moorfields Eye Hospital, London, UK) as part of a wider research project studying changes in suppression and visual function during conventional treatment for childhood amblyopia (to be reported elsewhere). The research was carried out in accordance with the tenets of the Declaration of Helsinki, and was approved by the UK Health Research Authority (REC ID #18/SC/0700; IRAS ID #248985).

### The “pCSF” Test

**Figure 1:**
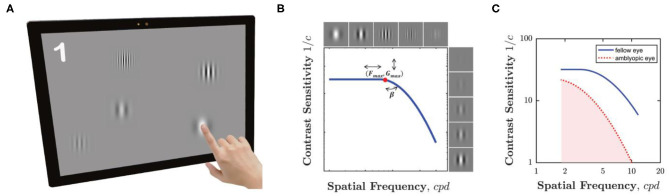
The pCSF test. **(A)** Hardware. Participants pressed equiluminant Gabor patches of variable frequency and contrast as they bounced around the screen. The maximum of five Gabors is shown here for effect, but typically only one or two (or zero) were displayed at any one time, and often some Gabors were suprathreshold. **(B)** Psychophysical algorithm. Stimulus selection and model fitting was performed using a Maximum Likelihood (QUEST+) algorithm, which attempted to fit the three-parameter model shown graphically here (and described formally in Equation 1). **(C)** Example CSFs for a single child aged 5.2 years, for both their amblyopic eye (red dashed line) and fellow eye (blue solid line). The shaded region indicates the area under the contrast sensitivity function (AUCSF) summary measure. A higher AUCSF value indicates greater overall contrast sensitivity.

Our novel “pop contrast sensitivity function” (pCSF) test is shown in Figure 1A. Users were simply required to “pop bubbles” (circular Gabor patches), by touching them as they “bounced” around a tablet screen. This was a truly gamified procedure (i.e., not a cosmetic wrapper for a conventional psychophysical test), although it was a fairly rudimentary implementation, which included relatively few audiovisual features and was primarily coded over a single weekend. The Matlab source-code for the pCSF test is freely available at: https://github.com/petejonze/pCSF.

### Hardware

The hardware consisted of a Microsoft Surface Pro 4 tablet, featuring a 12.3 inch, 2,736 × 1,824-pixel touchscreen display (Microsoft, Redmond, Washington, U.S.). This display is only 8-bit, so bit-stealing was used to obtain >10-bit luminance precision (23). The screen was calibrated (linearized) using central measurements from a CRS ColorCal Mk 2 colorimeter (Cambridge Research Systems, Cambridge, UK). It was not corrected for spatial non-uniformity. The screen's front-facing camera was used to perform real-time head pose estimation (see below), and ran at a spatial resolution of 640 × 640-pixels.

### Software

The test was programmed in Matlab 2016b, using Psychtoolbox v3 (24). Head tracking was performed by OpenFace 2.2.0 (25, 26), which is open-source software composed of compiled C++ code with various third-party dependencies, including OpenCV (27).

### Stimuli

The stimuli consisted of horizontal Gabor patches, presented against an isoluminant gray background (the mean {range} luminance across a 4 × 6 grid of uniformly spaced screen locations was 94.9 {88.1–102.4} cd/m^2^). Up to a maximum of five Gabors could be present simultaneously. On each frame, the probability of one new Gabor appearing was 1/60·N, where *N* was the number of Gabors already present and 1/60 represents the refresh rate of the screen. This meant that that the probability of a new Gabor appearing was inversely proportional to the number of Gabors already present. In practice, a new stimulus appeared on average every 3.1 s (median). During the test, each Gabor traveled independently across the screen, changing direction when reaching the screen edge or when touching another Gabor. Their direction and velocity was determined by a simple approximation of molecular dynamics (the Lennard-Jones potential, see source code for details). In practice median {IQR} velocity across all trials was 151 {104–191} pixels/sec. Phase and orientation were fixed at 0 and 90°, respectively. Michelson contrast and spatial frequency were free parameters, controlled by the psychophysical algorithm (*see below*). The range of spatial frequencies was limited such that every Gabor contained at least four visible bands, with a minimum of two pixels per band. The standard deviation of the Gaussian hull, *SD*_*px*_, was fixed at 52 pixels. At a nominal viewing distance of 50 cm, 52 pixels corresponds to 0.55° visual angle, meaning that 99% of stimulus energy fell within a diameter of 2.83°. For drawing purposes, the total spatial support (diameter) of each Gabor was 350 pixels. Each Gabor was visible for a maximum of 6 s (or until pressed), and to avoid hard temporal edges, the onset/offset of each Gabor was temporally ramped with 1-s cosine filters. The starting location of each Gabor was random, but constrained so that Gabors never overlapped or appeared outside the area of the screen.

### Task

A Gabor was considered “Hit” if the participant pressed within 221 pixels of its center (3$\cdot \sqrt{2}\cdot SD_{px}$) within 7 s of its onset (i.e., its 6 s visible duration, plus a 1 s grace period to allow for any ongoing motor responses to complete after stimulus offset). Stimuli not pressed within 7 s were removed and considered a “Miss.” Following each Hit, a “pop” sound played, and the Gabor was replaced with an image of a coin, 1 s in duration. To discourage guessing, a negative buzzer sound played after each False Alarm, though False Alarms were not entered into the psychophysical algorithm (see *next*). A running score was visible at the top left of the screen. This score began at zero, and increased/decreased by 1 after each Hit/False-Alarm (minimum: zero). There was no feedback or loss of points following a Miss. Note that the score was for motivational purposes only. These data are not reported, and because of the adaptive nature of the design all children would be expected to attain a similar score, irrespective of their CSF.

### Psychophysics

The core psychophysical algorithm (the “back end”) consisted of a QUEST+ (13, 14) (Maximum Likelihood) procedure, similar to the qCSF (7–12). It was the same algorithm that we have described in detail previously (6). However, in previous works it received input from a conventional four-alternative forced choice (4AFC) psychophysical task, whereas here the “front end” input was provided from the unconstrained, gamified procedure described above. In brief, the algorithm attempted to fit the 3 parameter model illustrated graphically in Figure 1B, and which is given formally by:

(1) $$\begin{array}{r} \alpha =\left\{ \begin{array}{cc} 1/{exp}_{10}\left({log}_{10}\left(G_{max}\right)-{log}_{10}\left(2\right){\left(\frac{{log}_{10}\left(f\right)-{log}_{10}\left(F_{max}\right)}{{log}_{10}\left(2\beta \right)/2}\right)}^{2}\right) & \text{if}f>F_{max}\\ {log}_{10}\left(G_{max}\right) & \text{otherwise} \end{array}\right., \end{array}$$

where *G*_*max*_ represents peak gain (contrast sensitivity), *F*_*max*_ peak spatial frequency, and β the rate of fall-off in sensitivity at high frequencies (full width half maximum, in octaves). Note that this formulation of the CSF represents a modified version of the log-parabola model recommended previously by Lesmes (11) and others (28). For simplicity, however, no fall-off at low spatial frequencies was included, allowing us to reduce the free parameters in our model to 3 (plus one for lapse rate, see below). This modification is unlikely to have had a substantive detrimental impact on the present results, since no stimuli below ~1.8 cycles per degree (cpd) were presented.

The stimulus domain consisted of 15 Michelson Contrast values log-spaced from 0.01 to 100%, and 10 spatial frequency values log-spaced from 0.019 to 0.125 cycles per pixel (1.8 to 11.7 cpd, assuming a nominal viewing distance of 50 cm). The parameter domain consisted of 15 *G*_*max*_ values log-spaced from 3 to 300; 10 *F*_*max*_ values log-spaced from 1 to 10; and 9 β values linearly spaced from 1 to 9: all with uniform priors. The underlying psychometric function was assumed to be a Weibull psychometric with a fixed slope of 3, a fixed lower asymptote (guess rate) of 0.05, and a variable (fitted) upper asymptote (lapse rate) of 0.05, 0.1, or 0.2. The model was updated after a Hit or a Miss (but not after a False Alarm).

Maximum likelihood algorithms are typically terminated after either a fixed number of trials, or when a given level of statistical confidence has been reached. As this was an initial feasibility assessment, however, the experimenter (author DE) terminated the test manually after the child had made ~30 correct responses. The medan {IQR} *N* trials, including misses, was 51 {43–63}.

### Analysis

Following standard practice, the final estimates of *G*_*max*_, *F*_*max*_ and β were computed as the mean of the QUEST+ posterior probability distribution. This distribution was refitted *post hoc* for greater fidelity. When doing so, the *G*_*max*_ and *F*_*max*_ parameter domains were increased to 40 elements each (NB: resulting in much larger search space, that could not have been processed in real time during the experiment itself). Furthermore, the spatial frequency stimulus domain was increased to 30 values log-spaced between 1 and 15 cpd. When performing this refitting, the spatial frequency of each Gabor patch was also recomputed, based on the presented stimulus value (in pixels), and the estimated viewing distance at stimulus offset, as estimated by OpenFace (see *next*).

### Head Pose Estimation

The location of the observer's head was monitored continuously by the tablet's front-facing camera, via OpenFace 2.2.0: a free machine-learning tool for facial landmark detection, head pose estimation, facial action unit recognition, and eye-gaze estimation (25). Estimates of viewing distance were made using a speed-optimized Convolutional Experts Constrained Local Model (CE-CLM). This yielding one vector of 〈*x, y, z*〉 location coordinates, in millimeters, per video frame. Estimates were made at ~29 Hz, although the sampling rate varied, depending on CPU availability. Note that OpenFace makes various assumptions in order to estimate viewing distance (e.g., regarding interpupillary distance). Ideally, these assumptions would be replaced by empirical measurements from each individual [or, alternatively, distance estimates could be calibrated by having observeørs wear/hold an object of known size; see Ref~(29)]. None of this was not done in the present work, however, as we wanted the test to remain as simple and pragmatic as possible. This may have contributed to random or systematic measurement error in the final CSF estimates.

Ideally, information regarding viewing distance would have been factored into the psychophysical algorithm live, during testing [i.e., as we have done previously when estimating visual fields (30)]. We did not do so here, however, as integrating live measurements is a non-trivial task (i.e., requires extensive pre-processing, and additional code), and if performed incorrectly can be counterproductive. This information was therefore factored in *post hoc*, based on estimated viewing distance at stimulus offset (i.e., at the point when the trial was scored a Hit or a Miss). Across all trials, median {IQR} estimated viewing distance was 531 {378–755} mm.

### Procedure

Testing was performed monocularly (once per eye), in a controlled research space. During testing, children wore their habitual best-corrected glasses, and the non-test eye was patched. The starting eye was randomized. In nine children (36%), the child's eyes had been dilated with tropicamide as part of their prior clinical appointment. However, the presence or absence of dilation did not appear to have any substantive impact on the results (see *Results*).

Children were not given any practice prior to testing, and were told simply to press any black and white stripes that they saw. Before the first trial, a tape measure was used to position the participant's head ~50 cm from the screen, and they were asked to keep their head still during testing. However, viewing distance was not strictly enforced, and children were observed to move considerably during testing (movements that were corrected for *post hoc* using head tracking). Note that 50 cm represents a tradeoff. Farther viewing distances allow higher spatial frequencies to be presented/tested (i.e., given the limited pixel density of the screen), and mean that a given head movement (in cm) has a relatively smaller effect on stimulus size (in degrees visual angle). Conversely, a shorter viewing distance (e.g., ~30 cm) would likely have been closer to the child's natural comfortable working distance (“Harmon distance”), and so may have reduced head movements. The choice of 50 cm was based on informal piloting, and may not have been optimal, particularly for young children.

Testing took place in a single session, and lasted no more than 10 min total. This testing took place after the child had completed a routine clinical appointment (~60 min), and after a further ~45 min of conventional psychophysical testing, as part of a wider research project (data collection ongoing). The pCSF test was the last task before children were discharged, and some of the younger children were visibly fatigued at this point.

Key outcome measures included: estimated contrast sensitivity (AUCSF), completion rates and test durations.

### User Feedback

Informal user feedback was obtained in two ways. First, the final 12 participants were asked to rate their enjoyment of the test, from 1 (“very low”) to 5 (“very high”). For reference, they were then asked to rate their enjoyment of a conventional psychophysical procedure performed earlier that day [specifically: a four-alternative forced choice visual crowding task: “Vac-Man,” described elsewhere previously (21)]. Further informal feedback was sought from the February 2020 meeting of the Moorfields Young Person's Advisory Group (“Eye-YPAG”: https://generationr.org.uk/eye-ypag). The Eye-YPAG is a group of older children (8–16 years), most of whom have first- or second-hand lived experience of various eye/vision conditions (not limited to amblyopia). Many members have experience of a wide range of clinical eye tests, and have taken part in clinical trials. During a 45-min session, these children were shown the pCSF test, and were invited to try it and provide unstructured feedback.

## Results

**Figure 2:**
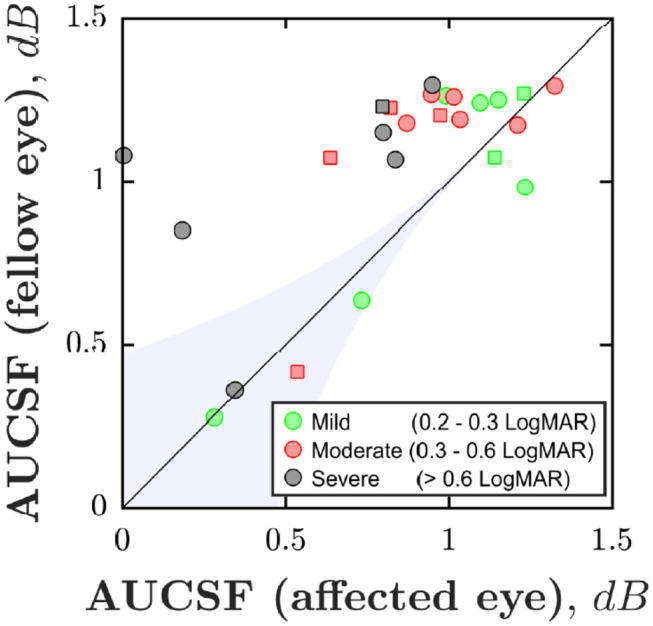
Case-control effect: Area Under the CSF (AUCSF) for each of the 25 individuals, broken down by eye. Marker color denotes amblyopia severity (as quantified by logMAR acuity in worse eye). Squares indicate cases of strabismus without anisometropia. The black line indicates unity (performance similar in both eyes). The blue shaded region of Figure 2 is for illustration only. However, points falling in a region such as this likely represent general non-compliance (poor performance in both eyes).

Figure 2 shows area under the CSF (AUCSF) scores for each individual. Ideally, all data points should fall above the unity line (i.e., indicating that the fellow eye is more sensitive than the amblyopic eye; “case-control effect”). This was the case for 100% of severe case, 70% of moderate cases, but only 50% mild cases (chance). Accordingly, the amblyopic eye was significantly less sensitive than the fellow eye in the moderate and severe cases, but not in the mild cases [*Wilcoxon signed-rank test for paired differences in AUCSF*; *P*_*mild*_ = 0.843; *P*_*moderate*_ = 0.028; *P*_*severe*_ = 0.016].

**Figure 3:**
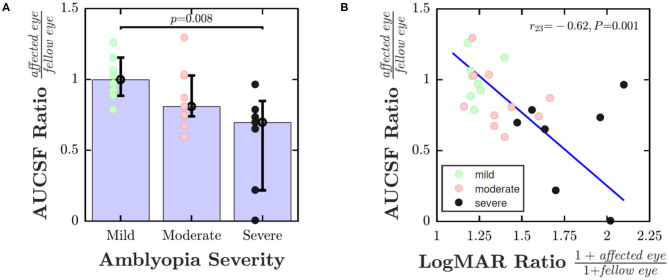
Dose effect: pCSF performance (ratio of affected eye AUCSF to fellow eye AUCSF) as a function of amblyopic severity. In **(A)** severity of amblyopia was categorized by logMAR acuity in worse eye (Mild: ≤ 0.3; Moderate: 0.3–0.6; Severe: > 0.6). In **(B)** severity of amblyopia was computed as the ratio of 1+logMAR acuity in the affected vs. fellow eye (+1 to ensure all values non-negative). Error bars indicate medians ± bootstrapped 95% confidence intervals. The line in **(B)** is the least-square geometric mean regression slope. See main text for details regarding statistics.

To explore whether a dose effect was also present, Figure 3 shows pCSF performance (ratio of affected eye AUCSF to fellow eye AUCSF) as a function of disease severity. One way of analyzing the data is to divide individuals into discrete severity groups (mild/moderate/severe), based on their logMAR acuity in their worse eye. This analysis is shown in Figure 3A, and indicated that children with more severe amblyopia had poorer pCSF performance than children with less severe amblyopia [*Kruskal-Wallis non-parametric one-way analysis of variance*; χ^2^ = 9.57, *P* = 0.008]. An alternative, more nuanced approach is to instead plot pCSF ratios against the ratio in logMAR scores between the two eyes. This more continuous approach is shown in Figure 3B, and gave qualitatively a similar result, with children with poorer (higher) logMAR ratios exhibiting poorer (lower) pCSF ratios [*Spearman's Rho*; *r*_23_ = −0.62, *P* = 0.001].

So far we have only considered AUCSF (a summary measure of overall contrast sensitivity). If the analysis in Figure 3B was instead repeated using each of the three individual CSF parameters in equation 1, no significant associations with amblyopia severity were observed (*P*_Gmax_ = 0.187; *P*_Fmax_ = 0.400; *P*_β_ = 0.464). The AUCSF effect was highly conserved, however, both in terms of significance and effect size, if we instead replaced AUCSF with a scalar measure of high-frequency cutoff (log sensitivity at maximum spatial frequency; *Spearman's Rho*; *r*_23_ = −0.62, *P* < 0.001). This may suggest that any differences in CSF were primarily related to changes at high spatial frequencies only; and/or it may simply reflect the fact that the reference variable (i.e., the x-axis in Figure 3B) is based only on logMAR acuity [i.e., and changes at low spatial frequencies may manifest independently (2–4)].

Median test duration did not significantly differ between the amblyopic and fellow eye [*Wilcoxon signed-rank test*; *Z* = 0.61, *P* = 0.545], and was 2.7 {*CI*_95_*:* 2.3–2.9} min for amblyopic eyes, and 2.5 {*CI*_95_*:* 2.2–3.1} min for fellow eyes. Across all eyes, median test duration was 2.6 {2.3–2.9} min.

The presence of pupil dilation (mydriatics) did not appear to affect performance, with no differences observed in mean test duration [*Wilcoxon ranked sum test*; *Z* = 0.08, *P* = 0.932], or in the AUCSF ratio [*Wilcoxon ranked sum test*; *Z* = 0.37, *P* = 0.713].

There was a small but significant difference in False Alarm rate between the two eyes [*Wilcoxon signed-rank test*; *Z* = 2.62, *P* = 0.009], with children more frequently pressing the screen incorrectly under their amblyopic (Median: 2.2 per min) vs. fellow eye (Median: 1.8 per min). In amblyopic eyes, the absolute number of False Alarms (which ranged from 1 to 25; Median: 5) also varied as a function of AUCSF [*Spearman's Rho*; *r*_23_ = −0.51, *P* = 0.009], with eyes with the lowest estimated sensitivity associated with the greatest number of False Alarms (note, False Alarm trials were not used when estimating sensitivity). There was, however, no association between estimated sensitivity (AUCSF) and the *total* number of screen presses (both correct and incorrect) [*r*_23_ = −0.09, *P* = 0.676], suggesting that children may have been attempting to maintain a relatively constant rate/number of responses between eyes.

**Figure 4:**
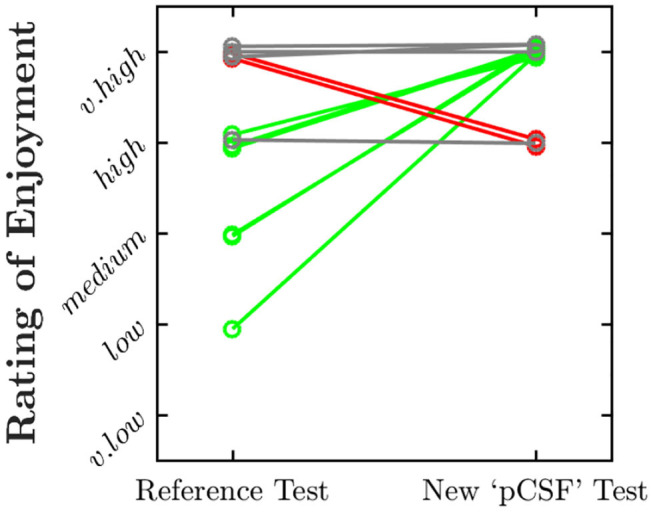
Paired ratings of enjoyment for the present “pCSF” test, and a more conventional psychophysical procedure. Values have been jittered slightly along the y-axis for visibility. Green and red lines highlight instances in which the child rated the present test as more enjoyable or less enjoyable, respectively.

As shown in Figure 4, all of the 12 children questioned rated the pCSF test as “enjoyable” or “very enjoyable.” Six children (50%) rated the test as more enjoyable than a conventional psychophysical procedure (Figure 4, green lines), although two somewhat preferred the conventional procedure, and four rated both equally highly.

The Eye-YPAG were generally positive in their assessment of the test. Several members noted that the test was more comfortable than conventional psychophysical procedures such as microperimetry, and that it was more fun and engaging than a letter chart. Some children remarked that the pCSF was actually somewhat boring, but much less so than current eye tests. The group recommended the future use of more varied feedback, sound effects, and some form of narrative in any future iterations of the test. Several individuals with nystagmus appreciated the lack of fixation cross. It was also noted that the pCSF is harder to cheat on than a static letter chart, where it is sometimes possible to memorize letters across repeat assessments.

In general, it was evident that none of the Eye-YPAG members had any difficulty comprehending what to do, and several individuals, when left to perform the test unsupervised, proceeded to reach a score of 100 or more (i.e., ~200+ trials): eventually having to be asked to stop.

Two potential issues of the current test were identified. Some individuals noted that, after extended use, smudges on the screen were liable to be mistaken for near-threshold stimuli. It was also noted that the current photopic background of the test made it unsuitable for individuals with photophobia (e.g., achromatopsia).

## Discussion

The results showed that a gamified, tablet-based test was able to produce plausible CSF estimates in a small cohort of young amblyopes (4.0–9.2 years). The test was able to separate moderately and severely amblyopic eyes from their fellow eye (case-control effect), and was able to distinguish between individuals with different degrees of visual impairment (dose effect). It was particularly encouraging that even the youngest children exhibited no difficulties completing the test or comprehending what to do, and in general, the children appeared to find the test genuinely enjoyable. This is particularly remarkable given that testing was performed after almost 2 h of clinical and psychophysical assessments, and given that the pCSF test itself was relatively rudimentary, with little in the way of sounds, graphics, or narrative. Conversely, we would hesitate to even attempt multiple CSF assessments in 4- or 5-year-olds using conventional psychophysical methods.

The test was far from perfect. It was insensitive to the effects of mild amblyopia. Furthermore, a minority of tests resulted in obviously spurious data, likely due to general non-compliance (e.g., low sensitivities in both eyes). These findings are to be expected given that the test was only a rough prototype, and also given the brevity of the test (which could have been allowed to run for longer).

Nevertheless, based on these preliminary findings, it appears that “gamified” vision assessments — such as the pCSF test described here — exhibit early promise as a potential means of estimating CSFs in young children: estimates which could in turn be used to identify, monitor, or stratify the severity of amblyopia.

At present, such functionality is provided by letter charts. However, digital tests could have substantial practical benefits by allowing vision to be measured outside of conventional eye-care facilities. Thus, for chronic conditions such as amblyopia, there is considerable interest in the idea of home monitoring, which has the potential to make treatment cheaper and more convenient by minimizing the number of in-person monitoring appointments (31). Home monitoring could also improve treatment outcomes by allowing for more frequent vision assessments (e.g., every few days or weeks, rather than every few months at present). This could be particularly beneficial for amblyopia given its low rates of treatment compliance (32, 33), and high rates of recurrence (34). Conventional tests such as letter charts are inappropriate for home monitoring, since a technician must be present to explain what to do, ensure the correct lighting and viewing distance, keep the child motivated and on-task, and record the results. In contrast, if, as in the present work, we can find tasks that children actually enjoy performing, and combine these with “smart” digital technologies (e.g., capable of monitoring viewing distance and ambient lighting autonomously), then home monitoring starts to become a realistic prospect. It is possible, for instance, to imagine a fully automated, cloud-based system in which the results of a digital test are transmitted securely to clinicians, who can then dynamically titrate or reinitiate patching remotely, or flag up high-risk cases for immediate, in-person review.

It is further possible that digital assessments of the whole CSF may be able to provide a more detailed and comprehensive characterization of visual impairment than conventional measures of acuity alone. For instance, previous studies have indicated that some amblyopes exhibit selective deficits at low spatial frequencies, independent of acuity (2–4). The present work is consistent with these previous findings, in that *G*_max_ (peak sensitivity) did not correlate with a conventional measure of amblyopic severity (based purely on acuity), and may therefore provide additional information not captured by acuity alone. In short, by measuring contrast sensitivity across a broad range of spatial frequencies, it is conceivable that pathologies such as amblyopia may be detected earlier, or monitored more robustly. Such benefits remain unproven, however, and at this stage we consider them secondary to the more practical benefits of digital assessment (detailed *above*).

### Limitations and Future Work

The present work represents a first step toward more engaging, child-friendly vision tests. However, it is only a first step: a preliminary assessment of feasibility. Further studies are required to formally assess the performance (sensitivity and specificity) of gamified measures, and to answer outstanding questions, such as whether gamified assessments are effective at sustaining a child's interest across repeated use, how robust they are when applied to a more diverse population (e.g., children with developmental delay), and whether the resulting data would be sensitive enough to detect changes in vision over time (e.g., due to treatment, or disease progression). These are questions that can only be answered by larger, longitudinal trials.

From a practical perspective, there are also myriad practical challenges to address before a test such as the one described in the present work could be made widely clinically available. These challenges include technical considerations (e.g., how to obtain more accurate estimates of viewing distance, how to factor these measurements into the psychophysical algorithm in real-time, how to store and transmit test data securely, and how to integrate the results with existing medical record systems), legal requirements (e.g., medical device certification), and issues surrounding usability and acceptability (i.e., among the patients themselves, their families, and also clinicians). Furthermore, even with a maximally engaging test, some instances of distraction or loss of concentration are inevitable. To achieve truly robust, unsupervised measurements will require autonomous means of verifying the user's identity, and of monitoring if/when they are performing the test correctly. These are non-trivial challenges, but ones that we have made initial steps toward solving using various computer vision and machine learning techniques (18, 35).

It may also be helpful in future to give further consideration to between-eye differences in response criterion. Thus, False Alarms were greater in the amblyopic eye, and tended to increase with severity. Put simply, children appeared disinclined to not press the screen for long periods, even when nothing was visible (note that while an adaptive algorithm would, given infinite trials, be expected to present the same proportion of visible stimuli to all eyes, in practice the algorithm always started from the same baseline stimulus level, and at some spatial frequencies amblyopic eyes might be at floor). The predicted effect of a more liberal response criterion would be to cause amblyopic severity to be *underestimated*. This may, however, have been offset in practice by the fact that the chance of a “lucky guess” was — in contrast to conventional n-alternative-forced-choice designs — relatively small (e.g., the probability of a random pixel/screen-press falling within any single Gabor was ~1%). I.e., and as the probability of a guess being correct tends toward zero, the deleterious effect of guessing becomes negligible. Nevertheless, given sufficient normative data it might in future be possible to “correct,” *post hoc* for the likely effect of response bias on performance (36). Furthermore, it may be prudent to display False Alarm rates as part of any test output, for general consideration by the assessing clinician (i.e., like when assessing visual fields via standard automated perimetry). Finally, the present work should not be taken to indicate that tried-and-tested psychophysical methods can easily be modified or replaced. For example, some clinical trials may benefit from the sorts of highly precise outcome measures that only a conventional psychophysical procedure can provide. While in older children, or in situations where the child *can* be manually supervised, the benefits of gamification may be negligible. Furthermore, it is important to note that even in the present study we focused only on one relatively successful approach to gamification (the pCSF test). During piloting, however, we also explored a range of other methods, many of which were unmitigated failures. This included, for example, one test in which the user is asked to “draw” their CSF directly, by tracing around striped parts of the screen (see Supplemental Text). Informal piloting (in non-naïve adults) indicated that this method was promising, and such as approach has been suggested previously as a potential, ultra-fast measure of the CSF (20, 37, 38). Children, however, seemed to find the task confusing — giving hesitant and highly variable responses — and the test was unable to differentiate between the two eyes, even in severe cases of amblyopia (see Supplemental Text).

## Summary and Concluding Remarks

The present work demonstrates the feasibility of using a truly gamified psychophysical procedure to measure spatial vision (the CSF) in amblyopic children. The pCSF test, which involved pressing equiluminant Gabor patches as they bounced around a tablet screen, and which used head tracking to control for changes in viewing distance, was appealing and intuitive to children, and exhibited promising, though imperfect, sensitivity. These preliminary findings suggest that there may be merit in developing such gamified procedures further, and in performing larger-scale investigations regarding their reliability, accuracy, adherence, and clinical utility. Such measures could be particularly valuable for assessing children outside of conventional eye-care facilities (e.g., home-monitoring or school screening).

## Data Availability Statement

The raw data supporting the conclusions of this article will be made available by the authors, without undue reservation.

## Ethics Statement

The research was carried out in accordance with the tenets of the Declaration of Helsinki, and was approved by the UK Health Research Authority (REC ID #18/SC/0700; IRAS ID #248985). Written informed consent to participate in this study was provided by the participants' legal guardian/next of kin.

## Author Contributions

PJ conceived the work, created the test materials, analyzed the data, and wrote the manuscript. PJ and DE designed the experiments. DE performed the experiment and collected the data. All the authors contributed toward finalizing the draft manuscript.

## Conflict of Interest

The authors declare that the research was conducted in the absence of any commercial or financial relationships that could be construed as a potential conflict of interest.
