# Supplementary material for: Can Psychophysics Be Fun? Exploring the Feasibility of a Gamified Contrast Sensitivity Function Measure in Amblyopic Children Aged 4–9 Years
Source: Front Med (Lausanne). 2020 Aug 26;7:469. doi: 10.3389/fmed.2020.00469 (PMC7480072; doi:10.3389/fmed.2020.00469)
Supplement: Supplementary file 1 [file Data_Sheet_1.PDF]

Supplemental Material

Can psychophysics be fun? Exploring the feasibility of a gamified Contrast Sensitivity Function measure in amblyopic children aged $4-9$ years.

Doaa Elfadaly[1,2], Sahar Torky Abdelrazik[2], Peter Thomas[1,3], Tessa Dekker[3,4], Annegret Dahlmann-Noor[1,3], Pete R. Jones[3,4,5*]

[1]*Moorfields Eye Hospital NHS Foundation Trust, London, UK, EC1V 2PD*

[2]*Department of Ophthalmology, Faculty of Medicine, Minia University, Minia City, Egypt, 61519*

[3]*NIHR Moorfields Biomedical Research Centre, London, UK, EC1V 2PD*

[4]*Child Vision Lab, Institute of Ophthalmology, University College London (UCL), London, UK, EC1V 9EL*

[5]*Division of Optometry and Visual Sciences, School of Health Sciences, City, University of London, London, England, EC1V 0HB*

[*]*Correspondence: Dr Pete Jones, Division of Optometry and Visual Sciences, School of Health Sciences, City, University of London, London, England, EC1V 0HB;* Peter.Jones@city.ac.uk

1. Further Pilot Data

In addition to the pCSF test reported in the main manuscript, a number of other tests were prototyped and explored as part of an extended period of research and development. One test that exhibited initial promise (in non-naïve adults) was the 'draw CSF' (dCSF) test, shown in **Figure S1A**.

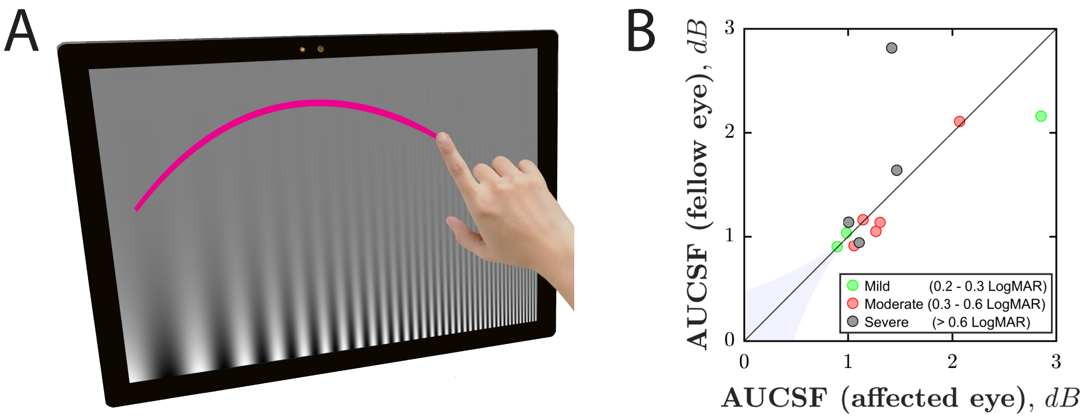

Fig S1. The 'dCSF' test, also trialled during piloting. **(*A*)** Method: children responded by using the touchscreen to trace their own CSF directly. **(*B*)** Pilot Data, shown in the same format as for the pCSF data reported in Figure 2 of the *main manuscript*.

In the dCSF test, a matrix of different spatial frequencies and contrast levels was displayed statically on the screen (i.e., a log-spaced swept frequency cosine signal in the horizontal dimension, with log-spaced contrast levels in the vertical dimension). Participants responded by simply using their finger to draw their CSF on the screen (i.e., tracing out the boundary of the smallest detectable contrast at each frequency, **Figure S1A** *magenta line*). In practice, the tablet only sampled touch inputs at $\sim$120 Hz, so the discrete array of sampled screen locations were interpolated/extrapolated by fitting a linear spline with 9 knots, uniformly spaced from left to right across the screen.

A complete CSF could be determined in mere seconds, though participants were free to subsequently modify/overwrite sections of the curve in order to refine the fit until satisfied.

To discourage and detect guessing, the horizontal direction of the stimulus was randomized (frequency ascending/descending) for each test, and the stimuli were shifted up/down by a randomly sized "blank" area running along the top side of the screen. A function was also developed to iteratively 'zoom in' on the region of interest by dynamically compressing the contrast range around the last inputted CSF (i.e., across successive stimulus presentations/swipes). In practice none of these features warranted further consideration, however, since it was evident from initial pilot results that the test gave poor results in young children.

The majority of children seemed to find the task confusing — giving hesitant and highly variable responses — and, as shown in **Figure S1B**, the dCSF test was unable to even differentiate the affected eye from the fellow eye.

At the time of writing, it is unclear whether this abject failure represents an intrinsic problem with the test (e.g., a lack of test-sensitivity due to observer criterion noise), or a more contingent problem with children not understanding what they were being asked to do – in which case it may be possible to refine the test by incorporating clearer instructions or practice trials (though at the risk of negating the primary appeal of the method: its speed).

What was clear, however, was that the dCSF was less intuitive and robust than the pCSF, so for the present study, we concentrated resources on developing and evaluating the latter.
